# Supplementary material for: Cyclin A triggers Mitosis either via the Greatwall kinase pathway or Cyclin B
Source: EMBO J. 2020 Apr 30;39(11):e104419. doi: 10.15252/embj.2020104419 (PMC7265243; doi:10.15252/embj.2020104419)
Supplement: Supplementary file 1 — Appendix [file EMBJ-39-e104419-s001.docx]

### APPENDIX

### for

### Cyclin A triggers Mitosis either via the Greatwall kinase pathway or Cyclin B

### Hegarat et al

### Appendix Table S1

### Antibodies used in this study

| **Antigen** | **Host** | **Company** | **Cat. Number** | **secondary ABs** | **Company** | **Cat. Number** |
| --- | --- | --- | --- | --- | --- | --- |
| **Alpha-tubulin** | Mouse | Abcam | nr. 7291 | Alexa647 donkey anti-rabbit | Invitrogen | A31573 |
| **Gamma-tubulin** | Rabbit | Abcam | ab84355 | Alexa555 donkey anti-goat | Invitrogen | A21432 |
| **Pericentrin** | Rabbit | Abcam | ab4448 | Alexa488 donkey anti-mouse | Invitrogen | A21202 |
| **CREST** | Human | ImmunoVision | HCT-0100 | HRP Goat anti-mouse | DAKO | P0447 |
| **Lamin A/C** | Goat | Santa Cruz | sc-6215 Lot I2111 | HRP Goat anti-Rabbit | Bethyl | A120-201P |
| **pLamin A/C S22** | Rabbit | Cell Signaling Technology | 2026S |  | | |
| **pCdk1-substrates** | Rabbit | Cell Signaling Technology | 9477S |  |  |  |
| **Aurora B** | Rabbit | Abcam | ab2254 |  |  |  |
| **KI67** | Rabbit | Abcam | ab16667 |  |  |  |
| **Cyclin A2** | Mouse | Abcam | ab38 |  |  |  |
| **Cyclin B1** | Mouse | Abcam | ab72 |  |  |  |
| **Cyclin B1** | Rabbit | Abcam | Ab32053 |  |  |  |
| **Cyclin B2** | Mouse | SC | sc-28303 |  |  |  |
| **Cyclin B3** | Mouse | SC | sc-515887 |  |  |  |
| **Cyclin B3** | Rabbit | ThermoFisher | PA5-37254 |  |  |  |
| **myc** | Mouse | Abcam | ab32 |  |  |  |
| **GAPDH** | Mouse | Genetex | GTX627408 |  |  |  |
| **Aurora A** | Mouse | Abcam | ab13824 |  |  |  |
| **Aurora B** | Rabbit | Abcam | ab2254 |  |  |  |
| **ENSA** | Rabbit | Abcam | ab180513 |  |  |  |
| **pENSA (ser67)** | Rabbit | CST | nr. 5240 |  |  |  |
| **Greatwall** | Rabbit | Sigma | HPA02717 |  |  |  |
| **Greatwall pThr198** | Rabbit | Hegarat et al. (2014) Plos Genet. (10) 1531-1552 | N/A |  |  |  |
| **Tpx2** | Rabbit | Bethyl | A300-429A |  |  |  |
| **Repoman (CDCA2** | Rabbit | Sigma | SAB4500599 |  |  |  |
| **CenpA** | Mouse | Abcam | ab13939 |  |  |  |
| **CenpB** | Rabbit | Abcam | ab25734 |  |  |  |
| **Topo2B** | Mouse | BD Transd. Labs | nr. 611492 |  |  |  |
