# Supplementary material for: Cyclin A triggers Mitosis either via the Greatwall kinase pathway or Cyclin B
Source: EMBO J. 2020 Apr 30;39(11):e104419. doi: 10.15252/embj.2020104419 (PMC7265243; doi:10.15252/embj.2020104419)
Supplement: Supplementary file 2 — Expanded View Figures PDF [file EMBJ-39-e104419-s002.pdf]

Expanded View Figures

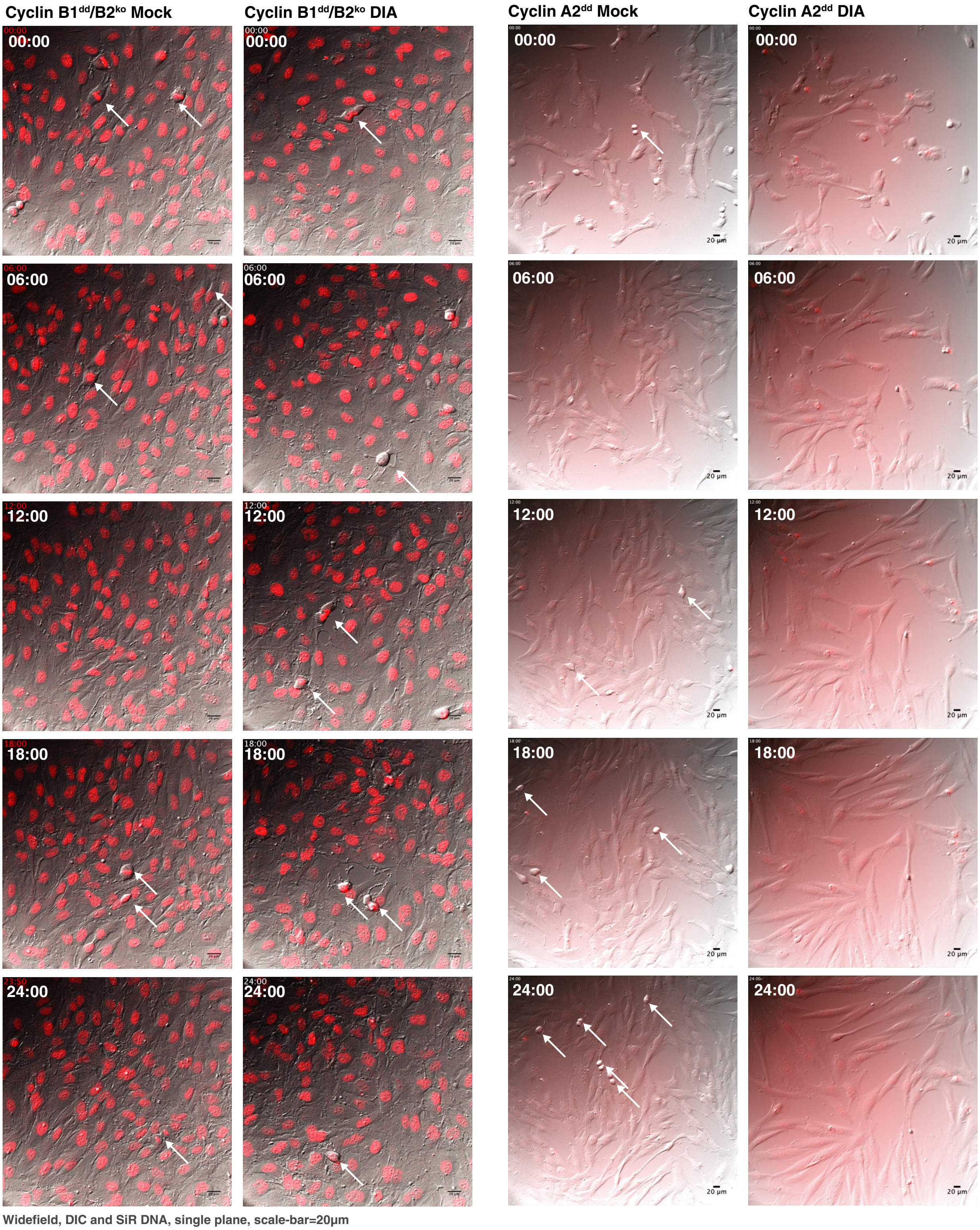

Figure EV1. Mitotic progression in RPE-1 cells lacking cyclins B1 and B2.

 © 2020 The Authors

Differential interference contrast (DIC) and fluorescence widefield video microscopy of mock or DIA-treated SiR-DNA-labelled cells with indicated genotypes. Images show a progression of 6 h time points (time indicated as hh:min, scale bar indicates 20 μm, and arrows point to dividing cells).

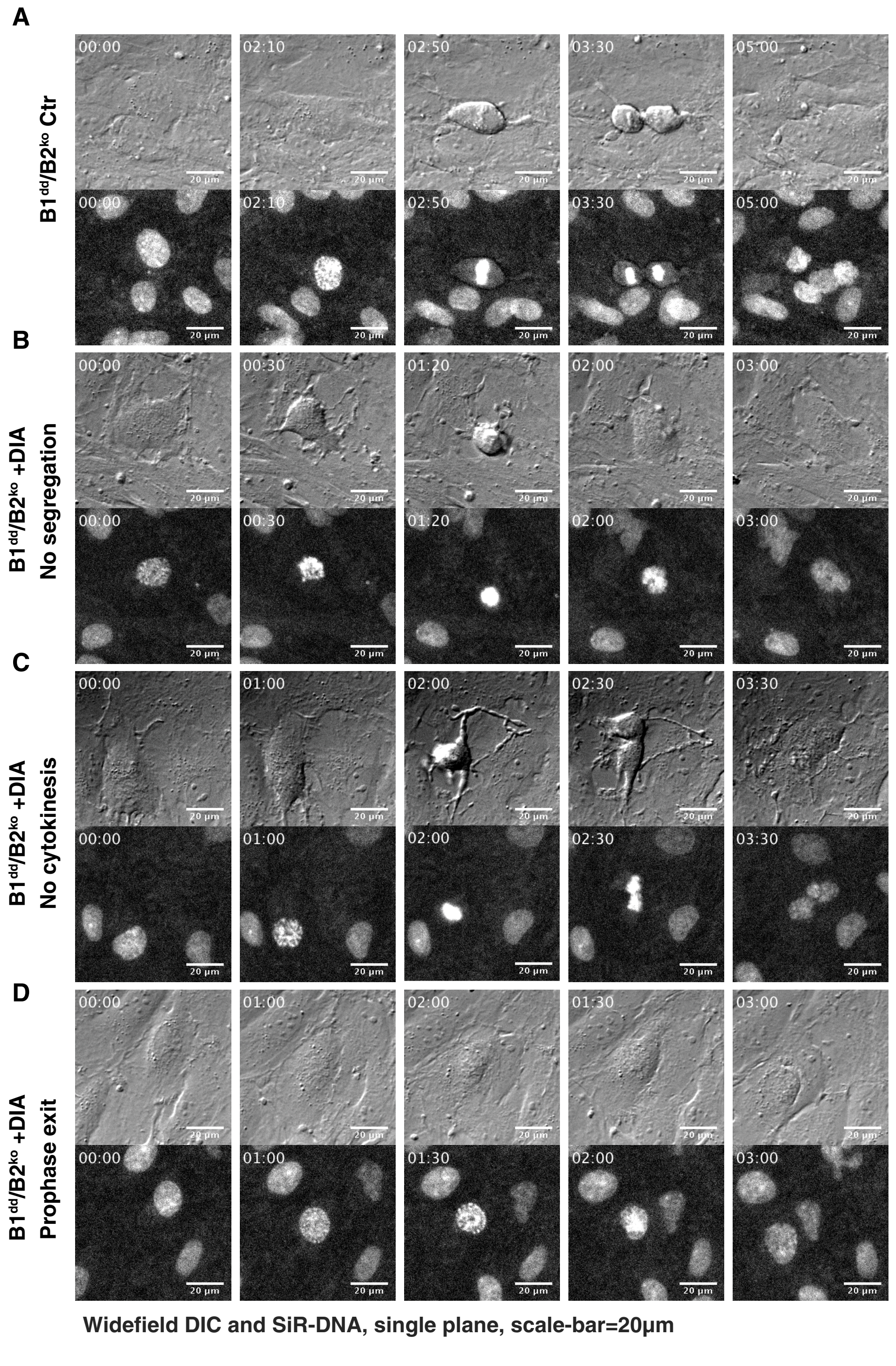

Figure EV2. Mitotic phenotypes of DIA-treated B1dd/B2ko cells.

Stills from live-cell imaging of asynchronously dividing B1^{dd}/B2^{ko} cells (DIC (top) and SiR-DNA (bottom), time is indicated as hh:min, and scale bar indicates 20 μm).

A A successful cell division in untreated B1^{dd}/B2^{ko} cells.

© 2020 The Authors

B DIA-treated B1dd/B2ko cells that fail to segregate sister chromatids and initiate cell division.
C DIA-treated B1dd/B2ko cells that attempt segregation and cytokinesis but fail to separate resulting in binuclear cells.
D DIA-treated B1dd/B2ko cells that fail to undergo NEBD and exit mitosis from prophase. All cells were imaged four to 6 h after DIA treatment.

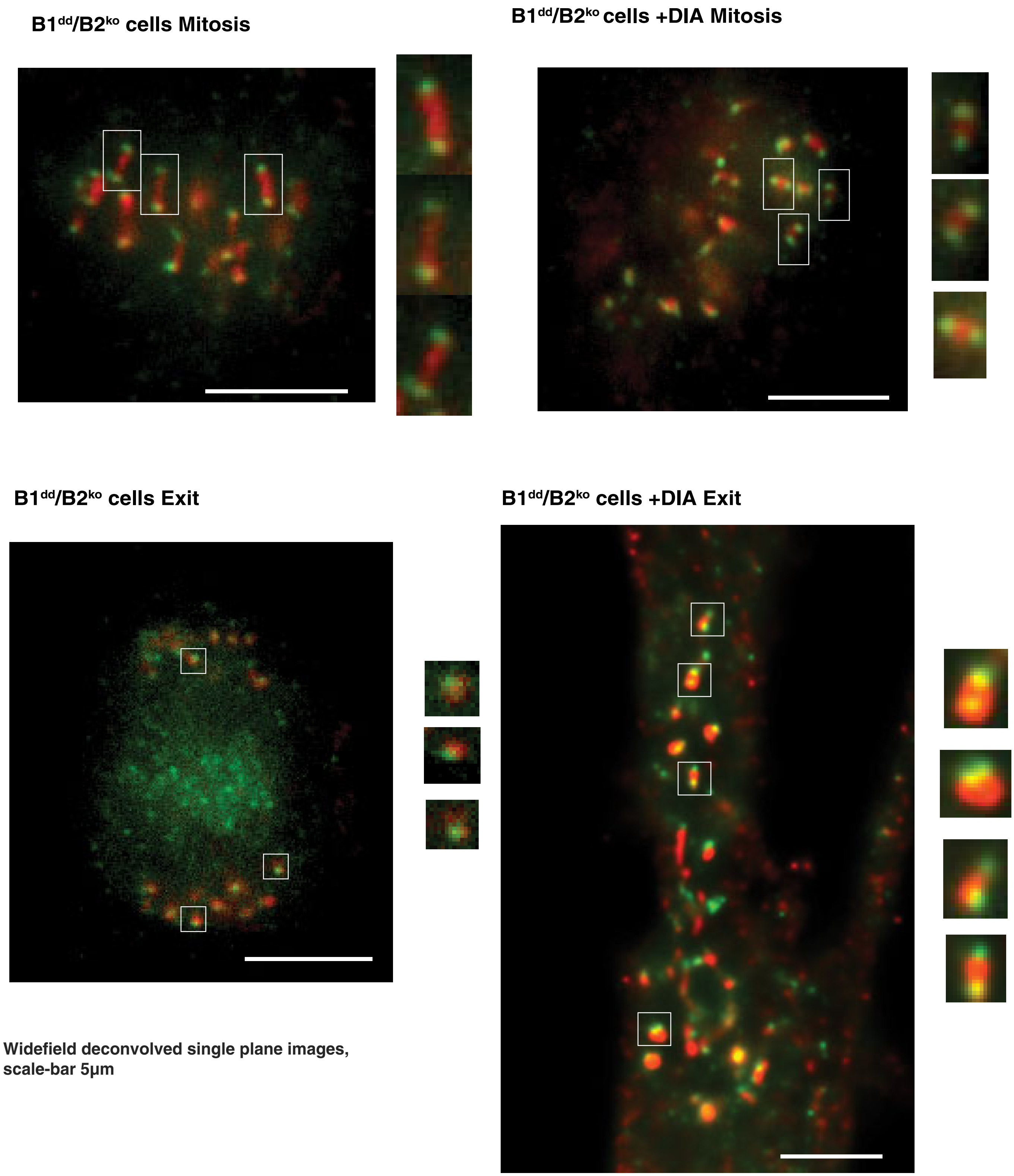

 © 2020 The Authors

Figure EV3. Analysis of Kinetochore tension and segregation.

Immunofluorescence images from mitotic (mitosis, top two panels) and ana/telophase (Exit, bottom two panels) Ctr- and DIA-treated $B1^{dd}/B2^{ko}$ cells stained with anti-CenpA (green) and anti-CenpB (red) antibodies. For DIA-treated cells, we determined the exit state by the onset of a cytokinesis furrow, since sister-chromatid segregation did not occur. Areas marked by squares are enlarged twofold at the right of each panel. The scale bar represents 5 μm.

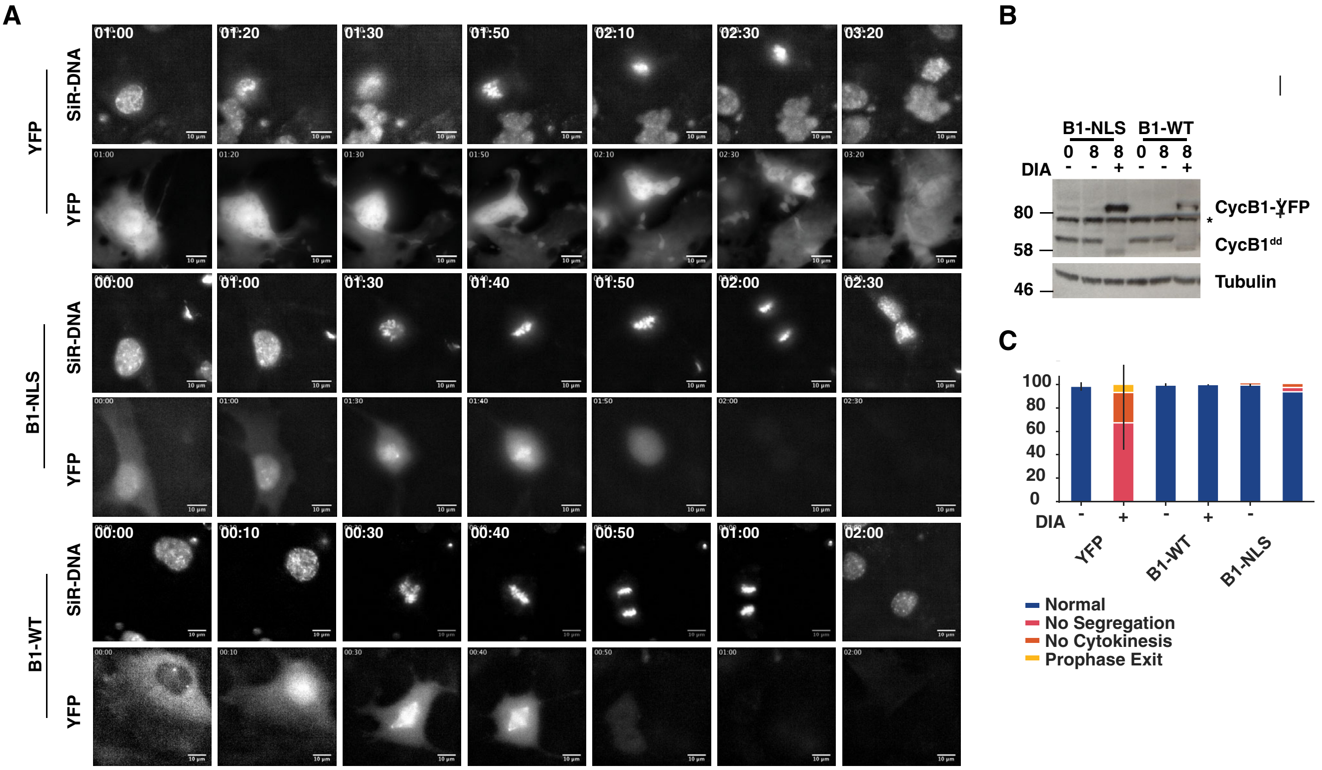

Figure EV4. Rescue of cyclin B phenotypes by induced expression of cyclin B.

A $B1^{dd}/B2^{ko}$ cells containing indicated inducible constructs were released from a single Thymidine arrest and treated with DIA to deplete endogenous cyclin B1 and induce expression of YFP, CycB1-YFP (B1-WT) or CycB1-YFP-NLS (B1-NLS). Imaging (SiR-DNA and YFP) was initiated 10 h after the release. The images displayed are from the time-lapse sequence at the indicated time points in hh:min. The scale bar represents a length of 10 μm.

B B1-WT- and B1-NLS-inducible cell lines were analysed for DIA-induced cyclin B1 induction/depletion. Samples were collected at indicated time points and probed by immunoblotting with cyclin B1 and α-tubulin antibodies (* indicates non-specific band).

C Quantification of the DIA-induced cell division phenotypes following induction of YFP, CycB1-YFP and CycB1-YFP-NLS induction. Data were collected from three independent experiments (n = 50), and the error bars indicate standard deviation between the three data sets.

© 2020 The Authors

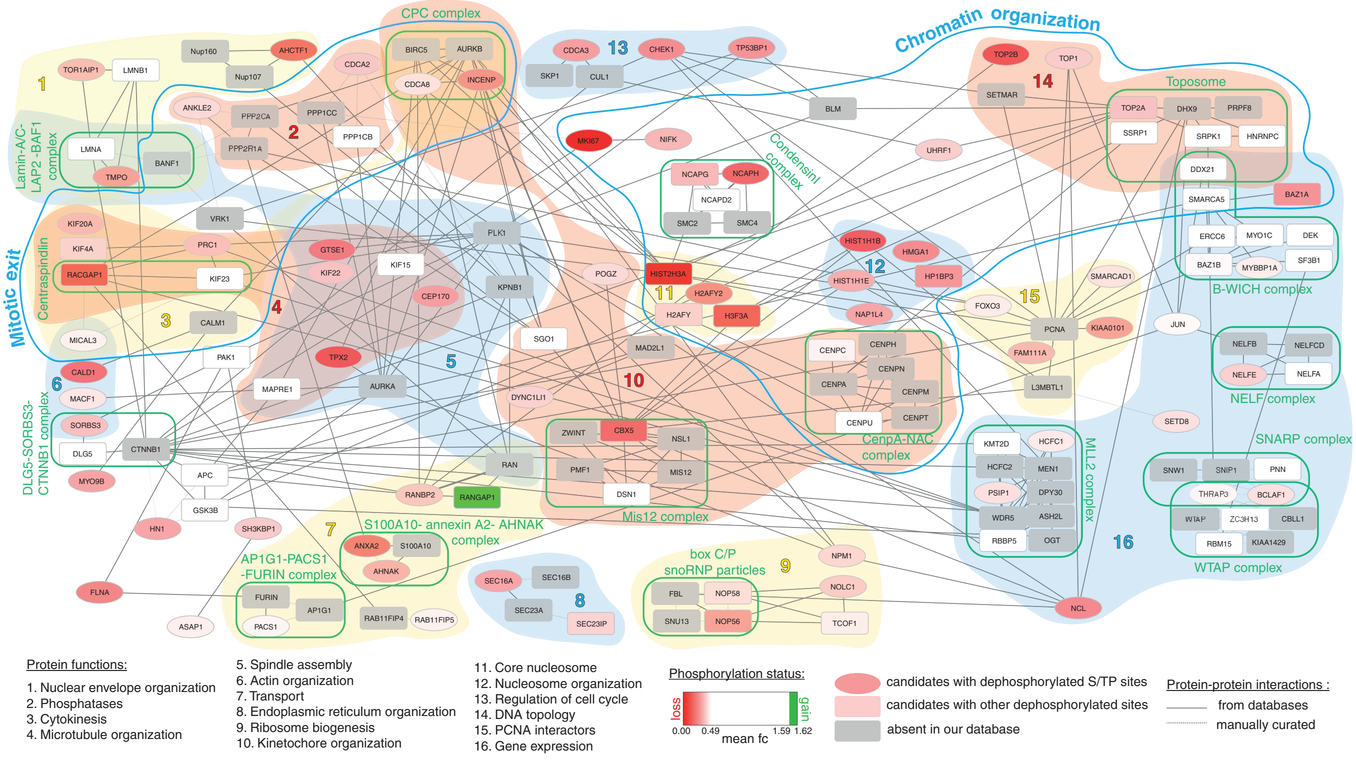

Figure EV5. Network analysis of identified cyclin B substrates.

Candidates with increased phosphorylation following cyclin B depletion are shown in green and candidates with decreased phosphorylation in red. Grey signifies proteins that are in a known complex, but absent from our data. S/TP sites are shown as ellipses.

 © 2020 The Authors
