# Supplementary material for: Cyclin A triggers Mitosis either via the Greatwall kinase pathway or Cyclin B
Source: EMBO J. 2020 Apr 30;39(11):e104419. doi: 10.15252/embj.2020104419 (PMC7265243; doi:10.15252/embj.2020104419)
Supplement: Supplementary file 11 — Code EV1 [file EMBJ-39-e104419-s011.docx]

### Appendix Computer Code S1

### XPPAut code for simulation mitotic entry on Figure 9

### CycA' = kscyca - kdcyca*CycA

### CycBT' = kscycb*(1-CycBT)*CycBT

### Wee1' = (kawee1' + kawee1*PP2AB55)^N*(1 - Wee1) - \

### (kiwee1'*CycA + kiwee1*Cdk1)^N*Wee1

### Cdc25P' = (kaCdc25'*CycA + kaCdc25*Cdk1)^N*(1 - Cdc25P) - \

### (kiCdc25' + kiCdc25*PP2AB55)^N*Cdc25P

### Cdk1' = (kacdk1' + (kacdk1"-kacdk1')*Cdc25P)*(CycBT - Cdk1) - \

### (kicdk1' + (kicdk1"-kicdk1')*Wee1)*Cdk1

### Gwlp' = (kaGwl'*CycA + kaGwl*Cdk1)*(1 - Gwlp) - kiGwl*PP2AB55*Gwlp

### pENSAt' = kpEnsa*Gwlp*(ENSAtot - pENSAt) - kdpEnsa*(B55tot - PP2AB55)

### PP2AB55' = (kdiss + kdpEnsa)*(B55tot - PP2AB55) - kass*PP2AB55*(pENSAt - (B55tot - PP2AB55))

### pSearly' = (kpearly'*CycA + kpearly*Cdk1)^M*(1 - pSearly) - kdpearly^M*pSearly

### pSinter' = (kpinter'*CycA + kpinter*Cdk1)*(1 - pSinter) - kdpinter*PP2AB55*pSinter

### pSlate' = (kplate'*CycA + kplate*Cdk1)*(1 - pSlate) - kdplate*PP2AB55*pSlate

### init CycA=0, CycBT=0.01, Wee1=1, Cdc25P=0, Cdk1=0, Gwlp=0, pENSAt=0, PP2AB55=0.25, pSearly=0, pSinter=0, pSlate=0

### # Values of kinetic parameters

### # for Cyclin synthesis & degradation

### p kscyca=0.02, kdcyca=0.02, kscycb=0.1

### # for Wee1 & Cdc25 activation &inactivation

### p kawee1'=0.2, kawee1=8, kiwee1'=1, kiwee1=2, N=2

### p kaCdc25'=1, kaCdc25=2, kiCdc25'=0.2, kiCdc25=8,

### # for Cdk1 activation & inactivation

### p kacdk1'=0.01, kacdk1"=1, kicdk1'=0.01, kicdk1"=1

### # for Greatwall, ENSA & PP2A:B55

### p kaGwl'=0.15, kaGwl=0.5, kiGwl=10

### p ENSAtot=1, kpEnsa=6, kdpEnsa=3

### p B55tot=0.25, kass=3600, kdiss=0.4

### # for phosphorylation & dephosphorylation of mitotic substrates

### p kpearly'=1, kpearly=7, kdpearly=0.05, M=5

### p kpinter'=0.1, kpinter=10, kdpinter=5

### p kplate'=0, kplate=1, kdplate=100

### @ xp=time, yp=CycA,xlo=0,xhi=100,ylo=0,yhi=1, total=100, meth=stiff

### @ nplot=8, yp=CycA, yp2=CycBT, yp3=Cdk1, yp4=pENSAt, yp5=PP2AB55, yp6=pSearly, yp7=pSinter, yp8=pSlate

### done
